# Nanomaterials for Inner Ear Diseases: Challenges, Limitations and Opportunities

**DOI:** 10.3390/ma15113780

**Published:** 2022-05-25

**Authors:** Liling Li, Jia Luo, Xuexin Lin, Jingqian Tan, Peng Li

**Affiliations:** 1 Department of Otolaryngology Head and Neck Surgery, The Third Affiliated Hospital of Sun Yat-sen University, No.600 Tianhe Road, Guangzhou 510630, China; lllctr@163.com (L.L.); 15626201730@163.com (J.L.); linxx67@mail2.sysu.edu.cn (X.L.); 2 Department of Otolaryngology Head and Neck Surgery, The Eighth Affiliated Hospital of Sun Yat-sen University, Shenzhen 518033, China; tan_jing_qian@163.com

**Keywords:** nanoparticles, intratympanic injection, hearing loss, inner ear, cochlear implants

## Abstract

The inner ear is located deep in the temporal bone and has a complex anatomy. It is difficult to observe and obtain pathological tissues directly. Therefore, the diagnosis and treatment of inner ear diseases have always been a major clinical problem. The onset of inner ear disease can be accompanied by symptoms such as hearing loss, dizziness and tinnitus, which seriously affect people’s lives. Nanoparticles have the characteristics of small size, high bioavailability and strong plasticity. With the development of related research on nanoparticles in inner ear diseases, nanoparticles have gradually become a research hotspot in inner ear diseases. This review briefly summarizes the research progress, opportunities and challenges of the application of nanoparticles in inner ear diseases.

## 1. Introduction

### 1.1. Treatment of Inner Ear Diseases

**Figure 1:**
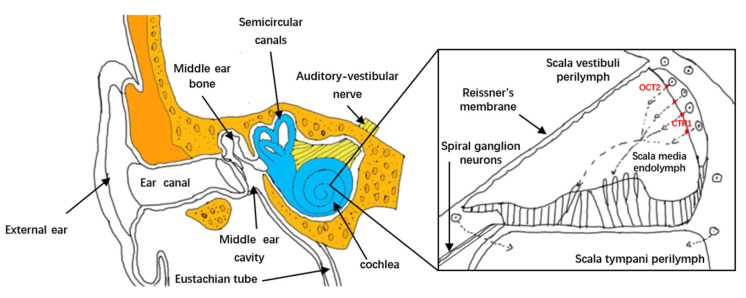
Inner ear anatomy. The most critical microvascular network in the cochlea is located in the cochlear stria vascularis. This vascular network receives a larger portion of the cochlear blood flow. The microvascular bed in this area also constitutes a tightly controlled BLB, rich in pericytes, and provides timely oxygen and nutrients for the cochlea. Cisplatin enters the cochlea from the bloodstream mainly through capillaries in the stria vascularis. Cisplatin may enter the endolymph through organic cation transporter 2 (OCT2) and copper transporter 1 (CTR1) of marginal cells.

There are three main treatment methods for inner ear diseases: systemic intravenous injection, tympanic membrane injection and surgical treatment. The biggest obstacle is the inner ear anatomy (as shown in Figure 1), especially the blood labyrinth barrier (BLB). These treatments can increase the retention time of the drug and the permeability of the round window membrane (RWM) or the oval window (OW) [1] (Table 1). The use of nanoparticles to assist local or systemic administration is a more feasible option, with the former having minimal side effects. At present, local inner ear drug delivery technologies have been developed, such as microneedle introduction [2], nanoparticles and hydrogels as carriers (as shown in Table 2). In recent years, nanoparticles have been widely used in sensing, image enhancement and transmission [3]. At the same time, applications in inner ear diseases are becoming more and more widespread, mainly because nano-delivery systems can encapsulate various therapeutic agents (drugs, proteins or genes), overcome anatomical obstacles, deliver them to target cells and even organelles and improve BLB biodistribution in tissues [4,5].

### 1.2. Nanoparticles Transport Mechanism

Currently, there are two main ways of local administration through the inner ear: tympanic membrane and cochlea (OW route and RWM route). Small lipid nanoparticles traverse the RWM via the paracellular pathway and then are engulfed by small bodies due to endocytosis, while larger nanoparticles are internalized by giant cells through a transcellular pathway. When the nanoparticles are internalized into the lysosome, they are trapped, which may lead to the release of the drug from the nanoparticles or the degradation of the nanocarrier. TEM imaging [7] showed that most of the platinum nanoparticles were absorbed by the cells into the lysosomes but did not enter the nucleus and mitochondria of the cell line. This indicates that endocytosis or phagocytosis plays a key role in the uptake of platinum nanoparticles by mammalian cells. Nanocarriers can accumulate in the cells or connective tissue of RWM without reaching the tympanic membrane. Liposomes and polymers can be highly aggregated inside the connective tissue and the outer layer of RWM, respectively, and then the drug can be released from the nanocarrier accumulated in RWM [8]. Han et al. [9] proposed an autocatalytic targeting method based on nanotechnology to encapsulate lexiscan, a drug that can regulate BBB, in nanoparticles, thus enhancing BBB permeability. The efficiency of targeted delivery of chlorotoxin-anchored nanoparticles was improved by autocatalysis for the treatment of stroke. Anionic vesicles are nanostructures assembled by surfactants with opposite charges that can distinguish hydrophobic from hydrophilic molecules. Cyclodextrin (CD) can change solubility by replacing water molecules with hydrophobic molecules or groups. The addition of β-cyclodextrin induced monolayer vesicles to become smaller and more stable [10]. Therefore, the permeability and transmembrane mode of nanocarriers are not invariable. Instead, some drugs can be used to increase the permeability of nanoparticles or make them more stable and less likely to be degraded. This reinforces the importance of studying nanoparticles in the inner ear.

## 2. Application of Nanoparticles in Drug Carrier

The diameter of nanoparticles ranges from 1 to 1000 nm. The nanoparticle system has the advantages of high drug stability, long sustained release period and high drug bioavailability. It is easily controlled by factors such as pH and magnetic field and is convenient to use [11], so it is suitable as a drug carrier. Nanoparticle materials commonly used as drug carriers include Poly (lactic-co-glycolic) PLGA, magnetism, lipids, liposomes, polymers, hydroxyapatite and silica (Table 3). Nanoparticles approved by the FDA are mainly liposomes, polymers and nanocrystals, but there is a trend to develop more complex nanoparticles, including micelles and protein-based nanoparticles, as well as various metals and metal oxides nanoparticles used in clinical trials [12]. Nanoparticles can be used for drug delivery and cell-to-cell communication. Among them, exosomes, as vesicles of nanocarriers, play a vital role in the progression of the disease [13]. In addition, some studies [14] have shown that extracellular vesicles derived from auditory HEI-OC1 cells may also be useful nanocarriers for the delivery of anti-inflammatory drugs and pro-degradation mediators. Nanoparticles are used for drug delivery, mainly for the treatment and prevention of drug-induced deafness, noise-induced hearing loss (NIHL), tinnitus, etc. Nano-drug carriers have different mechanisms of action in inner ear diseases (Table 4).

### 2.1. Drug-Induced Deafness Treatment

Mostly, the treatment mechanism of drug-induced deafness is that nanoparticles carry therapeutic drugs or combine with deafness drugs to promote degradation. Ramaswamy [23] used prednisolone-loaded magnetic nanoparticles in a mouse model of ototoxicity. Magnetic nanoparticles are controlled by a magnetic field, enter through RWM and deliver therapeutic amounts of steroids to the inner ear. The degree of hearing loss depends on changes in the number of hair cells. The more hair cells that survive, the smaller the impact on hearing. It was observed that the cochlear hair cells of the saline control group were reduced by 72%, and the hair cells of methylprednisolone injected into the tympanic membrane were reduced by 33%. However, the hair cells in the basal area of the cochlea in the magnetic nanoparticle treatment group decreased by only 9%. This indicates that the delivery of prednisolone nanoparticles significantly protects the OHC in the basal cochlea region from the influence of cisplatin and relieves inflammation. Nanoparticles have greatly improved the efficacy of treating drug-induced deafness.

### 2.2. Noise-Induced Hearing Loss Treatment

Excessive production of reactive oxygen species and inflammation are two key causes of NIHL, which can lead to OHC damage. Currently, nanoparticle systems can be prepared to reduce active oxygen damage and inflammation, thereby treating and reducing NIHL. The passage of cisplatin into the cochlea is shown in Figure 1. Kayyali et al. [33,34] developed an MFNPs (targeted multifunctional nanoparticles) system loaded with JNK pathway inhibitor D-JNKi-1, which can control the binding of drugs to specific inner ear cell types through targeted peptides. This can reduce the hearing loss caused by cisplatin. The use of MFNP in this study enhanced the delivery of D-JNKi-1 to the apical region, which is essential for human speech perception. Nanoparticles greatly improve the targeting and treatment efficiency of inner ear therapeutic drugs.

### 2.3. Tinnitus Treatment

In the treatment of tinnitus, the study of Farrah et al. [41] showed that a potential nano-scale carrier can be used to enhance the non-invasive transmission of the outer ear, providing a method for the treatment of tinnitus. Nano-carriers provide convenience for various drugs to enter the inner ear.

## 3. The Role of Nanoparticles in Gene Therapy of the Inner Ear

The idea of nanoparticles for gene therapy includes using nanoparticles as a carrier to target drugs or genetic material to specific parts of the cochlea to treat clinical hearing loss [42], which can also extend the duration of action of therapeutic drugs. Nanoparticles can keep genes or drugs in the body and protect them from enzyme damage. Innovative methods include introducing specific genes into cells to produce proteins or compensate for abnormal genes or gene transfection [43].

Common approaches to gene therapy for the inner ear include viral therapy, surgical treatment and non-viral therapy. The former mainly includes adenovirus, lentivirus, etc. (Table 5). Surgical treatment is still in the exploratory stage because it is uncontrollable and risky. Non-viral therapies mainly include synthetic peptides, liposomes and polymer nanoparticles. Among them, nanoparticles can improve the transfection efficiency in the process of gene therapy and play a very important role in gene therapy of the inner ear. The biggest advantage of nanoparticles is that they can be adjusted according to our needs, which can make up for the defects of virus treatment and surgery to a greater extent. Common encapsulated gene nanoparticles used to treat inner ear diseases are shown in Table 6. At present, a variety of synthetic or natural polymer nanoparticles have been developed and used for genetic testing. They include PLGA, polyethyleneimine, dendrimers, dextran and the like. In cochlear organ culture, hyperbranched polylysine nanoparticles (HPNP) have been shown to be effective in transfecting SGN. Most polymers easily combine with DNA molecules to form multimers. Then, these cationic polymers interact with the negatively charged cell surface to promote the absorption of DNA into the cell and release the DNA into the nucleus for gene expression [44].

The application of nanoparticles in animal gene therapy has been relatively mature, but the application of nanoparticles in human gene therapy is still limited by the anatomical structure differences between rodents and human ears and the acceptable invasive degree in clinical surgery. Nevertheless, animal models are still useful in exploring various cell-specific genetic vectors, optimizing viruses, reducing their immunogenicity, improving transduction efficiency and regulating the duration of gene expression.

## 4. Application of Nanoparticles Tracer in Inner Ear

In the research of nanoparticles, the application of ligands for cell targeting and tracking is rarely studied. Different from fluorescent staining tracing, the former has a lower spatial resolution and poor tissue permeability and generally can only be used on the surface. The application of nanoparticles in the tracer can effectively evaluate the treatment and prognosis of inner ear diseases. The lack of cell-specific delivery may limit current use in certain clinical applications such as cell regeneration. The great potential of nanoparticles to target specific cell types suggests that the relationship of ligand and receptor interactions between nanoparticles and inner ear cells deserves further study [11].

The tracer effect of nanoparticles is very valuable for studying the distribution and effects of drugs (Table 7). Magnetic nanoparticles have been approved for clinical use in the United States and Europe. In the United States, three types of magnetic nanoparticles have been approved for use in patients. Two of them (Feridex and GastroMark) are used as imaging contrast agents. A third magnetic iron oxide nanoagent is a therapeutic agent for the treatment of iron deficiency anemia. In Europe, two types of magnetic nanoparticles have received CE mark approval: Endomag’s Sienna+ and MagForce AG’s NanoTherm particles [53]. By using the same magnetization direction between the brain tissue and the two permanent magnets of magnetic nanoparticles, the movement of nanoparticles in brain tissue can be studied [54]. Recently, using fluorescent tracer chitosan nanoparticles through an OW, an opening in the vestibule was discovered after injection in the tympanic cavity of a guinea pig. This can be used as a drug delivery system method for peripheral vestibular diseases, thereby providing the possibility of effective treatment [55].

The tracer effect of nanoparticles can be better used to evaluate the therapeutic effects of ototoxicity and hearing loss. Yang et al. [56] developed an in vitro RWM model to screen nanoparticles. They used nanoparticles to successfully deliver dexamethasone (Dex) to inner ear hair cells and analyzed the biodistribution of Dex in the inner ear. In addition, Yang et al. made an animal model of ototoxicity and found that its therapeutic effect was better than dexmedetomidine sodium phosphate without nanoparticles.

The application of nanoparticles in the tracer is of great help in the treatment, distribution and evaluation of drugs for inner ear diseases.

## 5. Application of Nanoparticles in the Biological Functional Interface Materials of Inner Ear Devices

The application of nanoparticles in the biological functional interface material of the inner ear device is mainly to provide auxiliary electrical stimulation for the cochlear implant (CI) to improve the bactericidal activity (Table 8). After the use of CIs, most deaf patients can only partially recover their hearing, which is mainly related to the anatomical difference between the CI electrode array and the auditory neurons. Applying carbon nanotubes as a coating material to the electrode array can increase the contact area of CIs, thereby improving its use effect. During the irradiation process, the thermal energy of the photons is converted, and the gold NPs can produce rapid heating and induce calcium transients in the cells. Some in vitro experiments [61] have shown that gold NPs successfully stimulate the neural activity of CIs and can electrically stimulate CIs to restore some functions in patients with severe hearing loss.

Nanomaterials can be used for cochlear therapy and transformed into drug delivery to the inner ear to treat relevant pathological tissues without damaging cellular structures [62]. A large number of antibacterial nanomaterials are being explored as a new strategy for the development of fungicides, especially metal NPs, which are effective against both gram-positive and gram-negative bacteria. Due to their wide range of antibacterial effects, AgNPs have been proven to be one of the most valuable antibacterial drugs for many pathogens. Silver ions can destroy the cell wall of bacteria, inhibit the binding of the active coordination bond of the enzyme and the electron-donating group, and ultimately lead to bacterial apoptosis. Experiments [63] show that biosynthetic negatively charged AgNPs and natural functional modification products chitosan are beneficial to improving the antibacterial effects of modification and lysine. The application of this hybrid nanocomposite material has opened up a new paradigm for the treatment of otitis media and the control of bacterial resistance. Ziabka [64] conducted research on middle ear prostheses rich in nano-silver, confirmed the efficacy of ear implants in clinical trials of 3 patients, and showed bactericidal effects. Research [65] found in vitro that LiNbO_3_ NPs have obvious immunomodulatory activity on inner ear cells, can increase the β-defensin of human inner ear epithelial cells and have direct antibacterial activity against pseudomonas aeruginosa. When using nanomaterials, piezoelectric vibrations are enhanced, which results in fiber-composite structures that will support the growth of human nerve-like cells, showing the promise of new devices for the inner ear.

## 6. Limitations of Nanoparticles

Nanomedicine has the advantages of small particles, free from anatomical constraints, high reactivity and strong adsorption capacity. The use of nanoparticles in inner ear diseases can improve the absorption and utilization of drugs, achieve efficient target delivery, extend the half-life of drug consumption and reduce harmful side effects on normal tissues. However, the interaction between nanomedicine and various molecules, cells and organs of the body is based on a series of complex interactions between particles and biological media. The size, shape, arrangement, surface charge distribution and surface chemistry of nanoparticles have become the key factors that determine the efficiency of the reaction between nanoparticles and target biological media. In addition, the application of nanoparticles in inner ear diseases is limited by the toxicity of the nanoparticles themselves, especially particle distribution, uptake rate and clearance rate (Table 3). To a certain extent, they can damage the body’s immune system. For example, studies have shown [69] that AgNPs can temporarily destroy the biological barriers of the external auditory canal skin, middle ear mucosa and inner ear skin, and when distributed to the middle ear, they can cause partial reversible hearing loss. This is related to the potential recruitment of macrophages in the cochlea and the regulation of inflammation-related TLR signaling pathways through the ubiquitin editing protein A20. AgNPs may endow striatal basal cells and spiral ligament fiber cells with macrophage-like functions and enhance the immune activity of Corti organ non-sensory supporting cells by up-regulating CD68, which may be related to the activation of TLR4. A20 and RNF11 play a role in maintaining cochlear homeostasis by negatively regulating the expression of inflammatory cytokines.

Due to the limitations of nanoparticles, surgery and systemic drug delivery are still the first-line options. At present, some studies [42,56] are optimizing nanoparticles in order to improve the bioavailability of nanoparticles in the inner ear while reducing the limitations of nanoparticles.

## 7. Summary and Future Perspective

There is no doubt that nanoparticles, if applied to the clinical treatment of the inner ear, will greatly change the treatment of inner ear diseases. These are helped by the fact that nanoparticles can be loaded with drugs or combined with deafness drugs to act as anti-inflammatory and antioxidant agents; carry genes into the inner ear to facilitate gene transfection; as tracers and imaging agents, study drug distribution and exercise and efficacy evaluation; auxiliary CI electrical stimulation, combined with antibiotics, play an antibacterial role. The encapsulation efficiency, transfection efficiency, labeling efficiency and use efficiency of nanoparticles have been greatly improved. All of these have laid a solid foundation for its extensive application in the local medicine of inner ear, stem cell therapy and CI, which will reduce the inflammation of the inner ear, improve the hearing of patients and relieve tinnitus and other symptoms.

Kim et al. [70] used NPs with a particle size of 150.0 ± 3.2 nm and packaging efficiency of 64.4 ± 2.0% to distribute in Poloxham and then convert into gel. No cytotoxicity was observed even at 4 mg/mL, but the vector significantly prolonged drug release and action time. Li et al. [71] prepared a novel non-viral vector (hyaluronic acid-modified polyethylene imine nanoparticles) for cochlear gene transfection, and the transfection efficiency of the basal membrane was up to 81.7 ± 4.71%, with no obvious toxicity to the basal membrane cells. Xu et al. [72] used superparamagnetic iron oxide nanoparticles to label mesenchymal stem cells to evaluate the therapeutic effect of sensory neural hearing loss and found that the nanoparticle labeling efficiency was up to 95% and can also supplement iron. Some nanoparticles (BaTiO_3_ and LiNbO_3_) [73] are biocompatible enough, on the one hand, to improve the efficiency of new implantable hearing devices without damaging vulnerable human cells and neurons.

Despite the progress that has been made, it is clear that with inner ear diseases, the targeted therapy of nano-drugs and the adaptability of the body still need to be improved significantly. In order to improve their delivery and overcome the problem of low targeting, it has been proposed to coexpose or functionalize NPs with various protein molecules. Coating NPs with protein molecules prevents them from being recognized by cell receptors. It prevents NPs from being phagocytic and thus prolongs their circulation in the human body. It is well known that some pathogens can use innate mechanisms such as toxin release to evade endocytosis and survive longer in the host. Based on such an approach, further development of NPs can be attempted. When nanoparticles come into contact with our body fluids, the biomolecules in our body fluids attach to the NPs to form protein layers. The formation of this substance alters the natural properties and functions of NPs in vitro and thus alters the absorption of NPs. At the same time, some drugs in the inner ear can also interfere with NPs and reduce their uptake. Biological interactions are often ignored when designing and considering DRUG delivery systems based on NPs. Therefore, it is important to consider the targeted identification of nanoparticles with receptors in organisms, the influence of the body fluid environment on nanoparticles and possible interactions with drugs in the inner ear in order to achieve effective delivery from nanoparticles to the inner ear. Research on the transport mechanism of nanoparticles in the field of medicine combined with drug synthesis can improve the clinical application of nanoparticles in the inner ear.

While nanoparticles face challenges in the field of inner ear diseases, we believe the future of nano-drugs for inner ear diseases is as bright as ever. As new technologies are developed, and new mechanisms are investigated to overcome the challenges we highlight in this review, there is no doubt that a threshold will be crossed for the efficacy of nanoparticle drugs, which will ultimately lead to great efficacy in the future.

## Figures and Tables

**Table 1:** Mechanism and challenges of inner ear treatment plan.

Treatment Plan	Mechanism	Challenge
Systemic administration	Increase the blood circulation time of the drug	BLB
Tympanic injection	Increase the permeability of RWM and OW; promote the diffusion of the drug in the inner ear; increase the residence time of the drug	Obstruction of RWM, OW and stapes; part of the liquid medicine is lost in the Eustachian tube [6]
Surgical intervention	Operate treatment directly in the inner ear	Inner ear anatomy

**Table 2:** Advantages and disadvantages of local delivery.

Local Delivery	Advantages	Disadvantages
Nanoparticles	Enhanced cellular uptake; targeted delivery of drug particles	WM Poor permeability; eustachian tube leakage
Hydrogels	Increase RWM contact time; reduce Eustachian tube leak	Inherent variation of RWM permeability
Microneedle puncture	Increased permeability of RWM and OW; intuitive; less traumatic	Leakage of eustachian tube

**Table 3:** The main types of nanoparticles delivered by the inner ear.

Type	Experiment	Size (nm)	Mechanism	Main Application	Limitations
PLGA	Cai [15] Wang [16]	150–300	as an important part of the mitochondrial delivery platform	reduce hearing loss caused by gentamicin	hydrolytic instability in aqueous suspension
Magnetic	Zou [17]	50–60	using magnetic resonance imaging to control	molecular imaging of the inner ear	increased possibility of air bubbles during injection
Lipid	Liu [18]	200	lipid composition, high absorption rate	low frequency hearing loss	dose-dependent cytotoxicity
Liposome	Lajud [19]	160	low degradation	NIHL	cytotoxicity, and greatly affected by size
Polymer	Gunday [20]	Less than 250	long sustained release period, high local concentration	antibacterial	requires oleic acid as a co-solvent
Hydroxyapatite	Calabrese [21]	14–17	as a scaffold that allows AgNPs to grow directly on the surface of Mg-HA	antibacterial effect and cytotoxicity studies	minor toxicity
Silica	Glueckert [22]	50	increased drug loading	hearing loss caused by cisplatin	toxicity

**Table 4:** The application mechanism of nanoparticles in drug carriers. (a) Experiments related to drug-induced deafness. (b) Experiments related to noise-induced hearing loss. (c) Tinnitus related experiments.

Experiment	Nanosystem Type	Mechanism
Ramaswamy [23]	Magnetic nanoparticles (a)	Magnetic nanoparticles loaded with prednisolone are delivered to the inner ear through the window membrane, releasing therapeutic amounts of steroids
Kuang [24]	PLGA (a)	SS-31 modified PLGA improved SS-31 peptide-conjugated geranylgeranyl acetone
Youm [25]	biocompatible nanoparticles (a)	Biodegradable nanocarriers loaded with siRNA are used to silence MAPK1 to resist cisplatin-induced ototoxicity
Kayyali [26]	superparamagnetic iron oxide nanoparticles (a)	Combines strongly with cisplatin to protect cells from cisplatin-induced ototoxicity
Dai [27]	PLGA (a)	Make protein drugs stay in the inner ear longer
Zhou [28]	biocompatible nanoparticles (a)	Mitochondrial targeted nanoparticles loaded with geraniol protect zebrafish models from gentamicin-induced widespread ototoxicity symptoms
Wang [29]	biocompatible nanoparticles (a)	A666 conjugated nanoparticles target the prestin receptor of outer hair cells (OHC) to combat the ototoxicity of cisplatin
Gu [30]	polymer-lipid hybrid nanoparticles (a)	Strongly binds to cisplatin and protects cells from cisplatin-induced ototoxicity
Wang [16]	Biodegradable polylactic acid-glycolic acid nanoparticles (b)	Maintain the integrity of mitochondria, prevent the occurrence of cell death, and support the hypothesis of mitochondrial targeted delivery for the treatment of hearing loss caused by aminoglycosides
Zhou [31]	Peptide-modified nanoparticles (b)	Promote lysosomal escape and mitochondrial accumulation; follow the classic endocytosis or autophagy pathway to internalize hair cells through dynein-dependent and independent pathways
Martin-Saldana [32]	Polymer nanoparticles based on intelligent synthesis of amphiphilic copolymers (b)	The down-regulation of caspase 3/7 expression reduces the release of IL-1β and the accumulation of intracellular ROS
Kayyali [33]	Targeted multifunctional nanoparticle (MFNP) (b)	Binding to specific inner ear cells through targeting peptides
Simoni [34]	MFNP system (b)	Same as above
Xu [35]	Nanosystem based on zeolite imidazole ester framework (b)	Slow release methylprednisolone
Li [36]	Genetically modified nanoparticles (b)	Reduce drug dosage; improve drug bioavailability
Wang [37]	Solid lipid nanoparticles	Increasing local drug concentration to treat acute acoustic stress cochlear injury
Zhao [38]	Berberine vector (b)	Targeted at ROS and used for OHC targeted therapy of NIHL
Jung [39]	Pluronic F-127 nanoparticles (b)	Reduce ROS levels; improve hearing loss
Schmidt [40]	Porous silica nanoparticles (c)	Provide long-term brain-derived neurotrophic factor (BDNF) to improve the survival rate of spiral ganglion neurons (SGN) in vitro
Farrah [41]	PC LCnps (c)	Enhances the non-invasive delivery of the outer ear

**Table 5:** Common methods of inner ear gene therapy.

Type	Experiment	Infected Cells	Advantages	Disadvantages
Adenovirus (AdV)	Wang [45]	Dividing and non-dividing cells	Carry larger genes (up to 14 kb)	Immune response
Lentivirus	Wang [45]	Non-dividing cells	Arry exogenous DNA up to 8 kb	Limited efficiency in transfecting hair cells
Sendai virus	Kurioka [46]	Fibroblasts and spiral ligament cells	High transgene Expression efficiency and low pathogenicity; strong gene expression ability; wide host range	Low permeability through RWM
Herpes virus	Luebke [47]	Some cells	Inhibit the growth of ear tumors	Immune system
Adenovirus-associated virus (AAV)	Lee [48]	Sertoli cells, spiral edges, spiral Ligaments and spiral ganglion cells	Longer gene expression; lower immune response and toxicity; more stable; generally preferred	Small size, gene load limited

**Table 6:** The role of nanoparticles in gene therapy.

Nano-Type	Loaded Gene	Experiment	Year	Inner Ear Disease
siRNA	siHes1	Du [49]	2018	Noise-induced deafness
Cationic lipid	Cas9-guide RNA-lipid complex	Gao [50]	2018	Hereditary deafness
Tumor penetrating nanocomposite	short interfering RNA targeting TNF-α	Ren [51]	2019	Primary vestibular schwannoma
HPNPs	DNA molecules	Delmaghani [44]	2020	Nuclear gene expression
Neutral lipid nanoparticles ssPalm	BDNF mRNA	Miwa [52]	2021	Sensorineural hearing loss and prevention of SGN degeneration

**Table 7:** Nanoparticles tracing effect.

Experiment	Nanoparticles Type	Results
Lam [57]	Porous silica	Study on the retention and distribution of neurotrophic factor-3 in the cochlea after topical nano-administration
Youm [58]	Ferrocene	Cochlear Biodistribution Study
Zhang [59]	HPNPs	Study on the distribution of sensory hair cells
Ding [60]	Fluorescent tracer chitosan	The effectiveness of RWM and the exploration of the OW path of NPs transmission

**Table 8:** Auxiliary electrical stimulation of CI by nanoparticles.

Experiment	Nano-Type	Mechanism
Leso [55]	Nanocrystalline diamond	Neuron migration and proper interface adhesion
Cai [66]	Micro-textured nanocrystalline diamond	Guide nerve growth and create a new neural network for independent electrical stimulation of CIs
Damnjanovic [67]	Gold	Stimulate action potential
Richardson [61]	Gold	Heat rapidly and induce intracellular calcium transients
Wise [68]	Silica	Maintain the survival of SGN
